# Caspase-11-mediated tubular epithelial pyroptosis underlies contrast-induced acute kidney injury

**DOI:** 10.1038/s41419-018-1023-x

**Published:** 2018-09-24

**Authors:** Zhen Zhang, Xinghua Shao, Na Jiang, Shan Mou, Leyi Gu, Shu Li, Qisheng Lin, Yipei He, Minfang Zhang, Wenyan Zhou, Zhaohui Ni

**Affiliations:** Department of Nephrology, Ren Ji Hospital, School of Medicine, Shanghai Jiao Tong University, Shanghai, 200127 China

## Abstract

Contrast-induced acute kidney injury (CI-AKI) is a serious complication in patients after administration of iodinated contrast media and is associated with a significant high risk for severe renal failure and death due to the wholesale necrosis of the tubules and interstitial inflammation. Pyroptosis is a form of programmed lytic cell death that is triggered by inflammatory caspases, but little is known about its role in tubular epithelial cell (TEC) death and contrast-induced acute kidney injury. Here we show that systemic exposure to contrast media causes severe tubular epithelial pyroptosis that is mediated by the inflammatory caspases, caspases 4/5 in human TECs, or the murine homolog caspase-11 in mice in vivo and in mouse TECs in vitro. Knockdown of caspase-4/5 preserved human TECs from cell death and reduced the release of mature IL-1β, and in caspase-11-deficient mice, contrast-induced acute kidney injury was abrogated, indicating a central role for caspase-11 in acute kidney injury. In addition, deletion of caspase-11 in TECs reduced Gsdmd cleavage, which is the key process for execution of pyroptosis. These results establish the requisite role of epithelial pyroptosis in contrast-induced acute kidney injury and suggest that epithelial inflammatory caspases are an important therapeutic target for acute kidney injury.

## Introduction

Acute renal failure, the most severe form of acute kidney injury (AKI), is a devastating clinical complication of contrast-induced AKI (CI-AKI), which is usually defined as a rise in serum creatinine (SCr) of ≥ 0.5 mg/dl (≥44 µmol/l) or a 25% increase from baseline value, assessed within 48–72 h after a radiological procedure without an alternative etiology^1^. CI-AKI is now the third most common cause of hospital-acquired renal insufficiency, with a mortality rate of >14%^2,3^. AKI is characterized by an exaggerated host-defense immune response and necrotic tubular epithelial cells (TECs) loss of function, and is also frequently the source of damage-associated molecular patterns (DAMPs), which is the stimulating and amplifying factors of renal tubular damage and inflammation^4^. A central pathogenic feature underlying AKI is the breakdown of renal tubular epithelial barrier due to widespread TECs necrosis^4^.

As we know the kidney is an important excretion organ and renal tubules are the major component of the kidney, and are vulnerable to a variety of injuries including hypoxia, toxins, and some drugs, which are freely filtered by renal glomeruli and excreted by the kidneys. The epithelial cells are thus invariably exposed to the toxic drugs such as contrast media, which are key for the pathogenesis of CI-AKI^3,5^. Thus, renal TEC damage or death occurs and DAMPs, released by dead TECs, triggers a host-defense immune response and release of proinflammatory cytokines such as interleukin (IL)-1β^4^. Moderate immune-inflammatory response in the kidney is an adaptive feature of the host-defense response that helps resolve inflammation to avoid AKI, whereas an exaggerated inflammatory response followed by severe tubules damage and epithelial cell death is ultimately responsible for the high mortality of CI-AKI^3,4,6^.

Pyroptosis is a programmed cell death that is distinct from apoptosis and necrosis in signaling mechanism^7^. Apoptosis is a non-lytic form of cell death and is initiated by caspases 2, 8, 9, and 10, and requires the effector caspases 3, 6, and 7^4^. Necroptosis, similar to pyroptosis, is a lytic cell death mediated by kinases such as RIPK3^8^. Although the molecular mechanisms of the three types of programmed cell death differ, it is likely that the tissue inflammation involves multiple programmed cell death pathways, depending on the type of the inciting stimulus and cell type. Recent reports find pyroptotic cell death contributes to TEC necrosis and interstitial inflammation, and TEC pyroptosis enhanced the kidney damage in a unilateral ureteral obstruction model^9–11^. Pyroptosis is involved in renal TEC death upon renal ischemia-reperfusion injury via the inflammatory caspase-11^12^. These studies showed that TEC incur pyroptosis, a form of programmed cell death resulting in rapid cell lysis, by activation of inflammatory caspase-11 in mice. Caspase-11 cleaves gasdermin-D (Gsdmd) and induces release of the active membrane pore-forming Gsdmd peptide (also known as p30), which leads to lytic death of cells by swelling^13–19^. Our previous study demonstrated that TEC manifested a pyroptosis-like morphology after treatment with contrast media^20^, but whether caspase-11−mediated pyroptosis contributing to CI-AKI remains to be experimentally addressed.

Inflammatory caspases 4/5/11-mediated pyroptosis has until now been primarily studied in macrophages or dendritic cells^13,14^. Their role in destroying the TEC through widespread epithelial cells death and pathogenesis of CI-AKI remains unknown. Here we tested the hypothesis that renal TECs are a primary target for pyroptosis via extracellular contrast media by the inflammatory caspases 4/5/11, and that TECs pyroptosis is required for the induction of CI-AKI.

## Results

### Contrast medium induces renal TEC pyroptosis via activation of inflammatory caspases

**Fig. 1:**
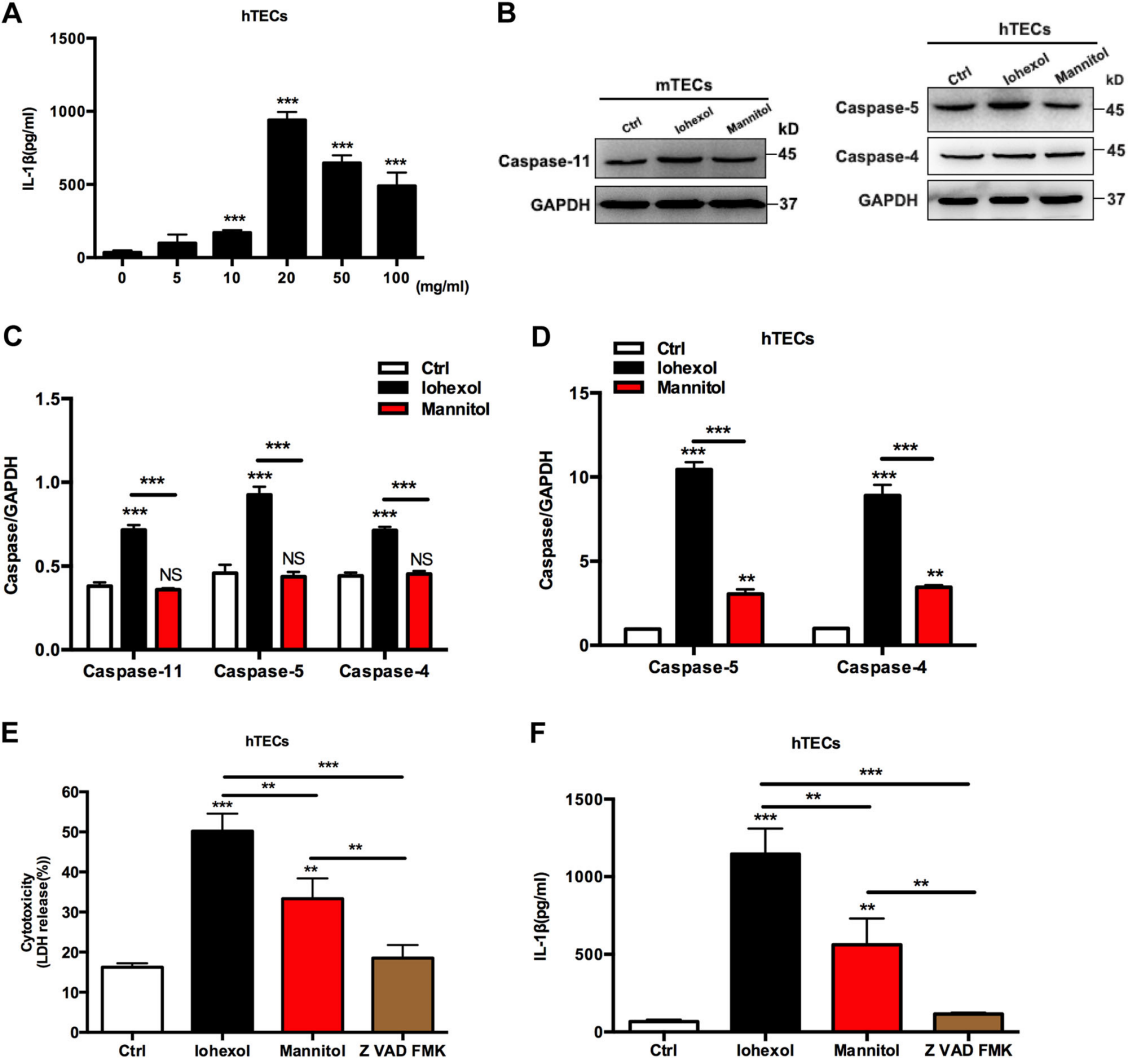
Iohexol induces TEC pyroptosis via activation of inflammatory caspases in mice and humans. **a** ELISA detection of mature IL-1β in human TEC culture supernatants 72 h after TECs treated with iohexol in a dose-dependent manner. **b** Immunoblot analysis of the inflammatory caspase-11 in mouse TECs and the inflammatory caspases 4/5 in human TECs with extracellular iohexol (20 mg/ml) or isosmotic mannitol for 72 h. **c** Quantification of the inflammatory caspases expression shows significant upregulation of caspase-11 and caspases 4/5 in mouse and human TECs, respectively. Statistics obtained from ANOVA. **d** mRNA expression of inflammatory caspases was determined by real-time PCR in human TECs after a 72 h period of iohexol (20 mg/ml) or isosmotic mannitol incubation. **e** Release of LDH showed that iohexol (20 mg/ml) or isosmotic mannitol incubation for 16 h led to marked cell lysis. Cell lysis was blocked by the pan-caspase inhibitor Z-VAD-FMK. **f** Incubation with iohexol (20 mg/ml) or isosmotic mannitol for 72 h increased mature IL-1β secretion, measured by ELISA in cell culture supernatants, whereas the response was abrogated by the pan-caspase inhibitor Z-VAD-FMK. Experiments were performed in triplicate. Ctrl, control. **P < 0.01, ***P < 0.001 vs. control or as indicated. Data are means ± SEM. Statistics obtained from ANOVA

We first addressed the question of whether TECs sense contrast media and initiated caspase-4/5/11-mediated pyroptosis. We used the low osmolar non-ionic monomer iohexol in the study. Release of inflammatory cytokines such as IL-1β is a main feature of pyroptosis and caspase-4/5/11 could activate the NLRP3 inflammasome, resulting in IL-1β cleavage^21,22^. We observed 72 h iohexol exposure-activated TECs to release IL-1β in a dose-dependent manner as low as 10 mg/ml (Fig. 1a). To identify the underlying mechanism, we next investigated the role of the inflammatory caspase-11 in mouse TECs and caspase-4/5 in human TECs. Caspase-4 is thought to be constitutively expressed in phagocytic cells, whereas caspase-5 is inducible^23,24^. We observed basal expression of caspase-11 in mouse TECs and caspase-4/5 in human TECs, and they were markedly upregulated by iohexol incubation for 72 h (Fig. 1b); the effect was more prominent with caspase-5 (Fig. 1c). Accordingly, significant gene upregulation was also observed in human TECs by iohexol (Fig. 1d). Thus, upregulation of caspases 4/5/11 was required for IL-1β maturation and secretion, and the activation of pyroptosis in TECs by contrast media.

Pyroptosis, also known as a regulated cell death, is characterized by cell lysis. Human TECs showed significant cell lysis after iohexol treatment, whereas the effect was blocked by the pan-caspase inhibitor Z-VAD-FMK (Fig. 1e), confirming that this was due to a caspase-mediated lytic cell death. Consistent with inhibition of human TECs lysis due to iohexol-induced upregulation of inflammatory caspases, we also observed that the maturation and secretion of IL-1β in human TECs by iohexol was obviously reduced (Fig. 1f).

### Caspase-4/5/11 is required for contrast-induced IL-1β cleavage and pyroptosis in mouse and human TECs

**Fig. 2:**
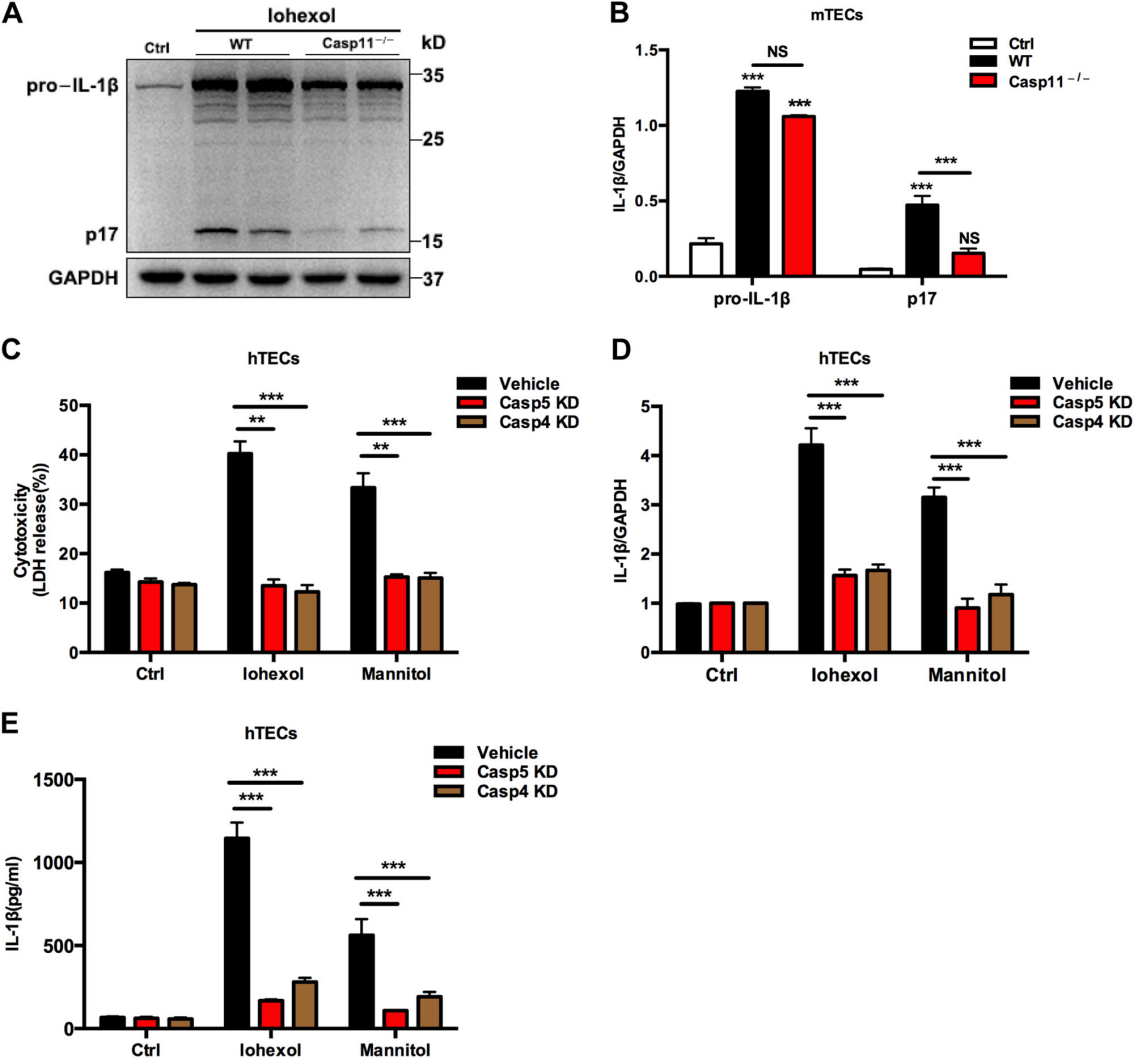
Requirement for caspase-4/5/11 in mediating contrast-induced IL-1β cleavage and pyroptosis. **a** Immunoblot analysis of the pro-IL-1β and mature IL-1β in TECs isolated from WT and Casp11^–/–^ mice treated with extracellular iohexol (20 mg/ml) for 72 h and the two duplicate samples represented separate study of TECs isolated from WT and Casp11^–/–^ mice treated with extracellular iohexol. **b** Quantification of the pro-IL-1β and mature IL-1β expression shows significant upregulation in TECs from WT mice, whereas the response in mature IL-1β was blocked in TECs from Casp11^–/–^ mice. LDH release by human TECs (**c**), mRNA expression of IL-1β determined by real-time PCR (**d**), and mature IL-1β in human TEC culture supernatants (**e**) after a 72 h period of iohexol (20 mg/ml) or isosmotic mannitol incubation were blocked by knockdown (KD) of the human inflammatory Casp4/5. Experiments were performed in triplicate. Ctrl, control; Casp 4/5, caspases 4/5; KD, knockdown. **P < 0.01, ***P < 0.001 vs. control or as indicated. Data are means ± SEM. Statistics obtained from ANOVA

IL-1β is a proinflammatory cytokine synthesized as a 31 kDa precursor protein and proteolytically activated by inflammatory caspases to release mature IL-1β (also known as p17) protein during pyroptotic cell death^13,14,17,25^. To address whether IL-1β cleavage is mediated by caspase-11, TECs were isolated from Casp11^−/−^ mice and wild-type (WT) mice and immunoblotting was performed for pro-IL-1β and mature IL-1β protein. TEC-specific deletion of caspase-11 prevented IL-1β cleavage following iohexol treatment for 72 h (Fig. 2a, b). To further investigate the role of the inflammatory caspase-4/5 in pyroptosis. We used caspase-4 and -5 small interfering RNAs (siRNAs) and observed that downregulation of caspase-4 or -5 by siRNAs prevented iohexol-induced cell lysis, maturation, and secretion of IL-1β in human TECs (Fig. 2c, d and e). Thus, caspase−4/5/11 is required for iohexol-mediated TEC pyroptosis.

### CI-AKI involves tubule damage, renal inflammation, and failure

**Fig. 3:**
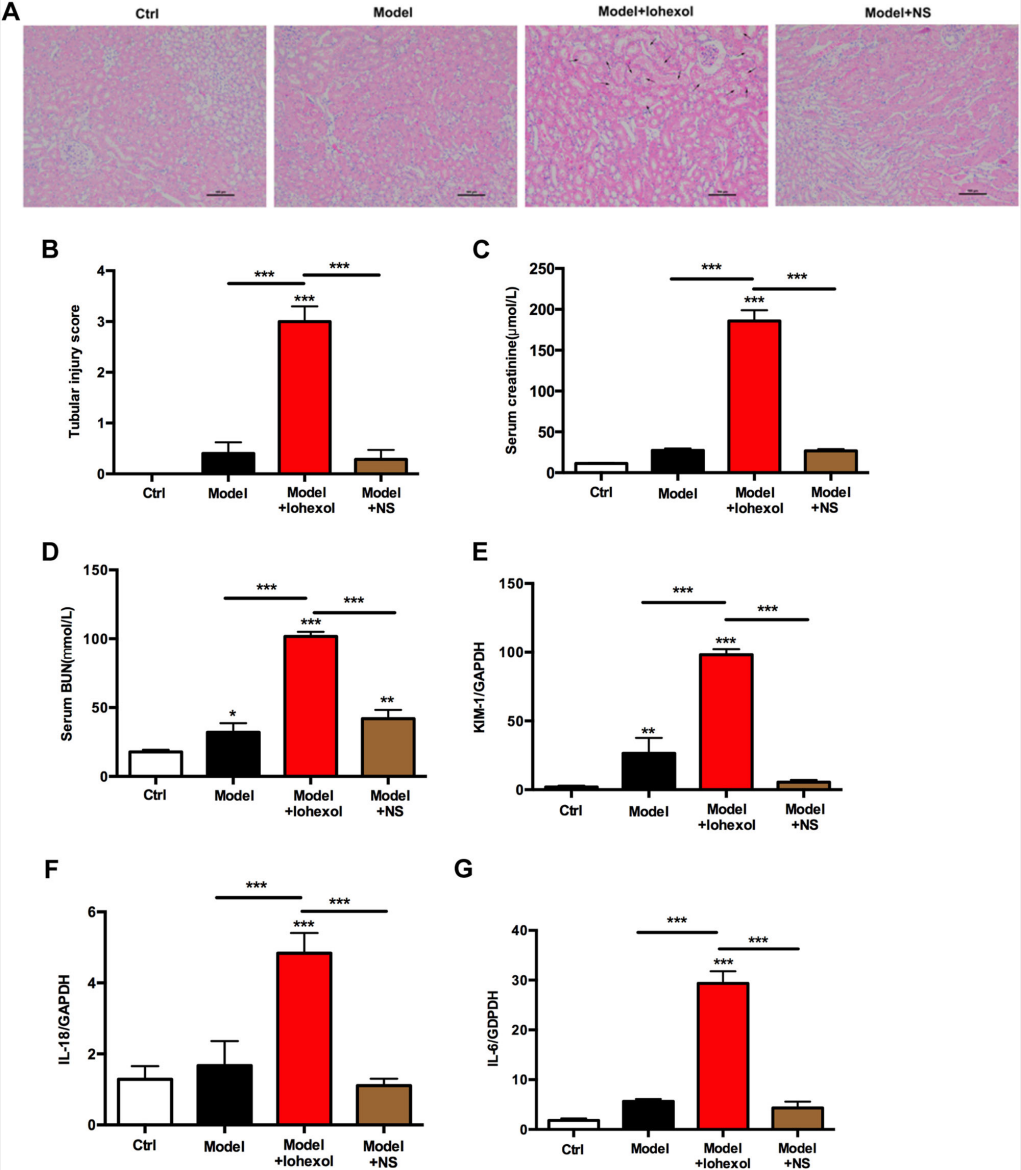
Contrast-induced acute kidney injury in C57BL/6 mice. **a** Representative H&E staining (*n* = 8 mice per group) of kidney sections from WT mice shows marked tubular injury (indicated by arrows) at 24 h following iohexol injection by tail vein (10 μl/g), whereas the effect was absent in the Ctrl, model mice, and mice injected with NS groups. Scale bars: 100 μm. **b** Quantitative analysis for tubular injury in **a**. Iohexol injection caused significantly increased SCr (**c**) and BUN levels (**d**), compared with that in the Ctrl, model, and model + NS groups. **e** Renal mRNA expression of markers of acute kidney injury and inflammation was determined by real-time PCR. BUN blood urine nitrogen, Ctrl control, NS normal saline, SCr serum creatinine, WT wild type. **P < 0.01, ***P < 0.001 vs. control or as indicated. Data are means ± SEM from five to six mice per group. Statistics obtained from ANOVA

To assess histologic features of tubular injury, kidney sections stained with hematoxylin and eosin (H&E) were examined in a blinded manner. Iohexol injection by tail vein could result in renal TECs injury with swelling and vacuolization, and interstitial inflammation within the outer medulla, as well as within the cortex, which were absent in the control, model mice, and mice injected with normal saline (NS) (Fig. 3a, b). The structural alterations of iohexol-induced AKI were associated with acute renal failure, as shown by a transient increase in SCr and blood urine nitrogen (BUN) levels, peaking at 24 h, compared with those in control, model mice, and mice injected with NS (Fig. 3c, d). Intrarenal mRNA expression of the AKI biomarkers KIM-1 and IL-18, as well as that of the proinflammatory cytokine IL-6, was increased after iohexol injection (Fig. 3e–g). Together, these observations indicated that murine CI-AKI was characterized by diffuse tubular injury mainly at the renal corticomedullary boundary zone, intrarenal inflammation, and acute kidney failure.

### CI-AKI is associated with caspase-11-mediated pyroptosis

**Fig. 4:**
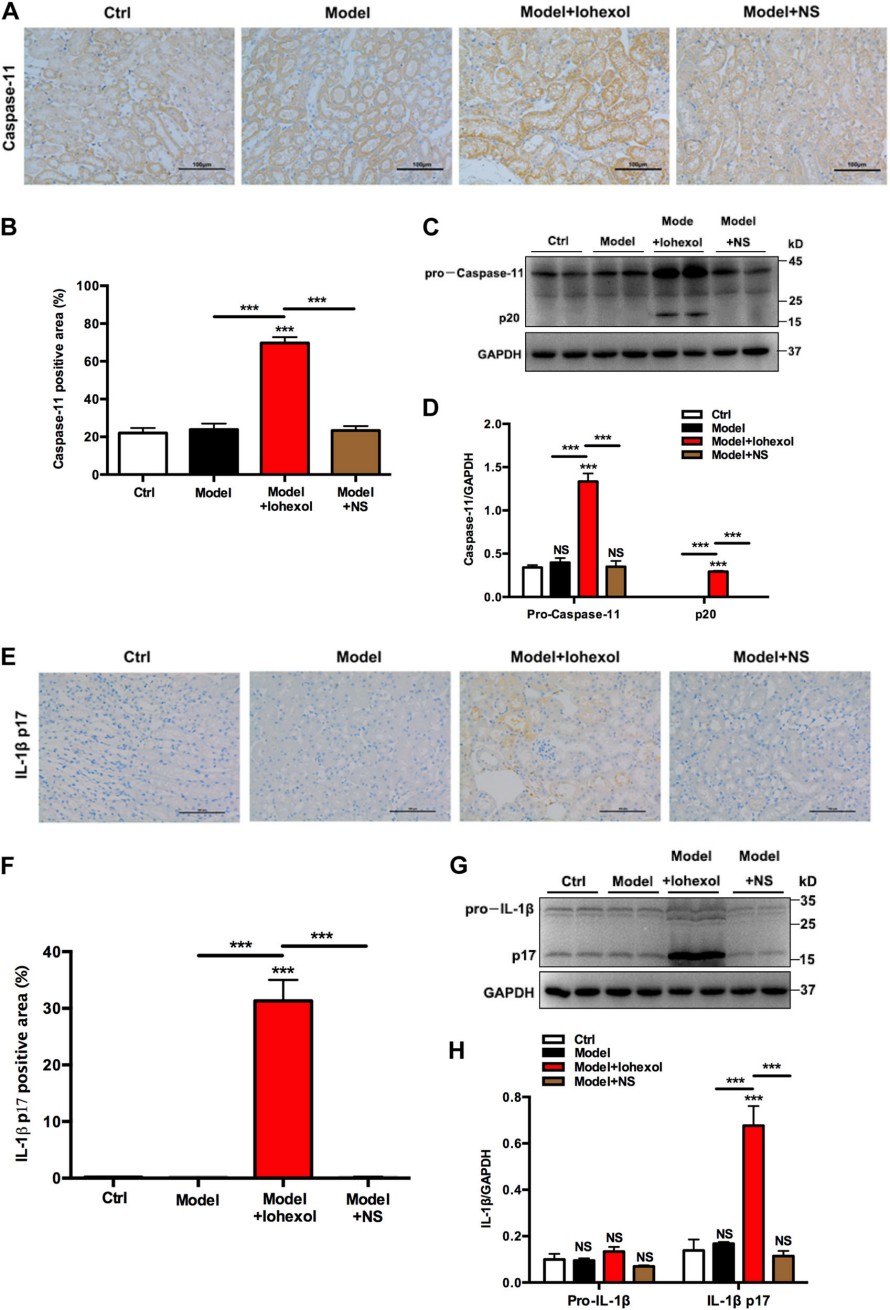
Involvement of Caspase-11 and IL-1β in contrast-induced acute kidney injury. **a** Representative images (six visual fields for each tissue sample analyzed) of immunolabelling for caspase-11 in the indicated experimental groups. Scale bars, 100 μm. **b** Quantitative analysis for caspase-11-positive area in the kidneys by ImageJ. **c** Immunoblot analysis of caspase-11 and cleaved caspase-11 (p20) showed that iohexol (10 μl/g) injection by tail vein markedly increased the expression of caspase-11 and p20, as compared with that in the Ctrl, model, and model + NS groups. **d** Quantification of the caspase-11 and p20 expression shows significant upregulation in WT mice injected with iohexol (10 μl/g), compared with that in the Ctrl, model mice, and mice injected with NS. **e** Representative images (six visual fields for each tissue sample analyzed) of immunolabeling for mature IL-1β in the indicated experimental groups. Scale bars, 100 μm. **f** Quantitative analysis for mature IL-1β-positive area in the kidneys by ImageJ. **g** Immunoblot analysis of pro-IL-1β and mature IL-1β showed that iohexol (10 μl/g) injection by tail vein markedly increased the expression of mature IL-1β, as compared with that in the Ctrl, model, and model + NS groups. **h** Quantification of the pro-IL-1β and mature IL-1β expression shows significant upregulation in WT mice injected with iohexol (10 μl/g), compared with that in the Ctrl, model, and model + NS groups. Renal tissue samples were collected at 24 h after injection of iohexol (10 μl/g) or NS via tail vein. Ctrl control, NS normal saline, WT wild type. ***P < 0.001 vs. control or as indicated. Data are means ± SEM from five to six mice per group. Statistics obtained from ANOVA

To evaluate the role of caspase-11 in the pathogenesis of CI-AKI, we first determined whether caspase-11 expression was upregulated in WT mice injected with iohexol. Immunological staining showed mice injected with iohexol showed a marked increase in caspase-11 expression, which was absent in control, model mice, and mice injected with NS (Fig. 4a, b). Immunoblot analyses of caspase-11 in the iohexol group also showed 3.9-, 3.3-, and 3.8-fold increases in the protein levels, respectively, compared with that in control, model, and mice injected with NS groups (Fig. 4c, d). We next investigated whether the pyroptotic marker IL-1β was involved in CI-AKI. Consistent with the increase in the expression of caspase-11, immunological staining and immunoblot analyses all showed that iohexol could significant increase the mature IL-1β expression in mice injected with iohexol, compared with that in control, model mice, and mice injected with NS (Fig. 4e–h). These results indicate that caspase-11-mediated pyroptosis has a vital role in CI-AKI.

### Caspase-11 is required for CI-AKI

**Fig. 5:**
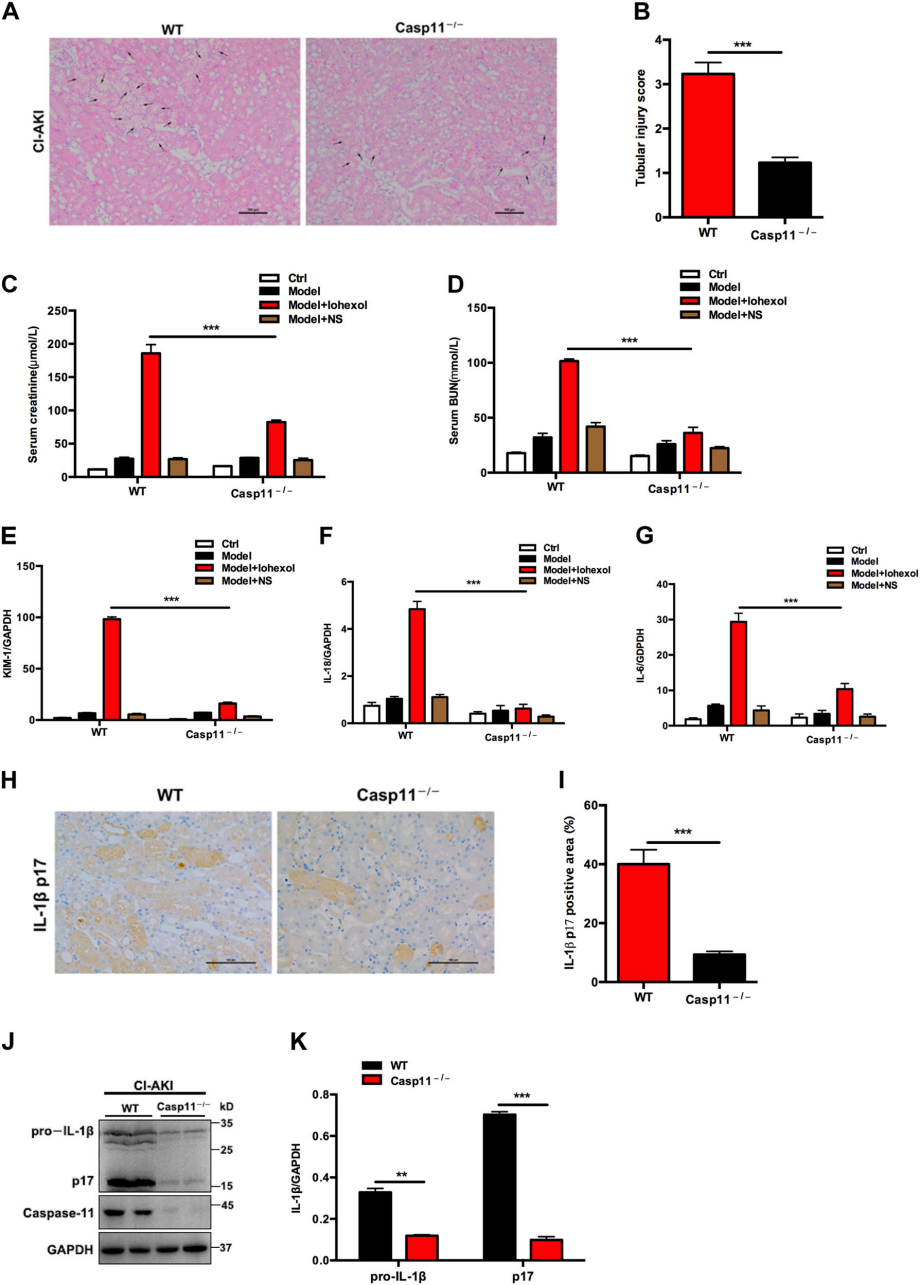
Caspase-11 is required for contrast-induced acute kidney injury. **a** H&E-stained sections of the kidneys from iohexol-exposed mice at 24 h shows tubular injury (indicated by arrows) in WT but not in Casp11^–/–^ mice. Scale bars: 100 μm. **b** Quantitative analysis for tubular injury in WT and Casp11^–/–^ mice. **c**, **d** Casp11^–/–^ mice showed decreased SCr levels and BUN levels. **e**, **f**, **g** Real-time PCR demonstrated that markers of acute kidney injury and inflammation were significantly downregulated in Casp11^–/–^ mice. **h** Representative images (six visual fields for each tissue sample analyzed) of immunolabelling for mature IL-1β in the indicated experimental groups. Scale bars, 100 μm. **i** Quantitative analysis for mature IL-1β-positive area in the kidneys by ImageJ. **j** Immunoblot analysis of pro-IL-1β and mature IL-1β showed that Caspase-11 knockout could markedly decreased the expression of mature IL-1β in mice injected with iohexol (10 μl/g), as compared with that in WT mice. **k** Quantification of the pro-IL-1β and mature IL-1β expression shows significant downregulation in Casp11^–/–^ mice injected with iohexol (10 μl/g), as compared with that in WT mice. BUN blood urine nitrogen, Ctrl control, NS normal saline, SCr serum creatinine, WT wild type. **P < 0.01, ***P < 0.001 vs. control or as indicated. Data are means ± SEM from five to six mice per group. Statistics obtained from ANOVA

We next further investigated whether CI-AKI was due to caspase-11 expression in the kidneys. Here we used Casp11^–/–^ mice to evaluate the tubular injury, intrarenal inflammation, and kidney function in CI-AKI. Histologic features determined by H&E in Casp11^–/–^ mice following exposure to iohexol (10 μl/g) demonstrated that deletion of caspase-11 could fully prevent iohexol-induced AKI in contrast with WT mice (Fig. 5a, b). Caspase-11 deletion also preserved the kidney function by significant decrease in SCr and BUN levels, compared with those in WT mice (Fig. 5c, d). Intrarenal mRNA expression of the AKI biomarkers KIM-1 and IL-18, as well as that of the proinflammatory cytokine IL-6, showed marked reduction following caspase-11 deletion, as compared with that in WT mice (Fig. 5e–g). Further, analysis for levels of mature IL-1β in the kidney, released during pyroptosis, also showed marked reductions following caspase-11 deletion (Fig. 5h–k). It demonstrated the central role of caspase-11 in mediating CI-AKI.

### Renal TEC caspase-11 activation is required for Gsdmd cleavage

**Fig. 6:**
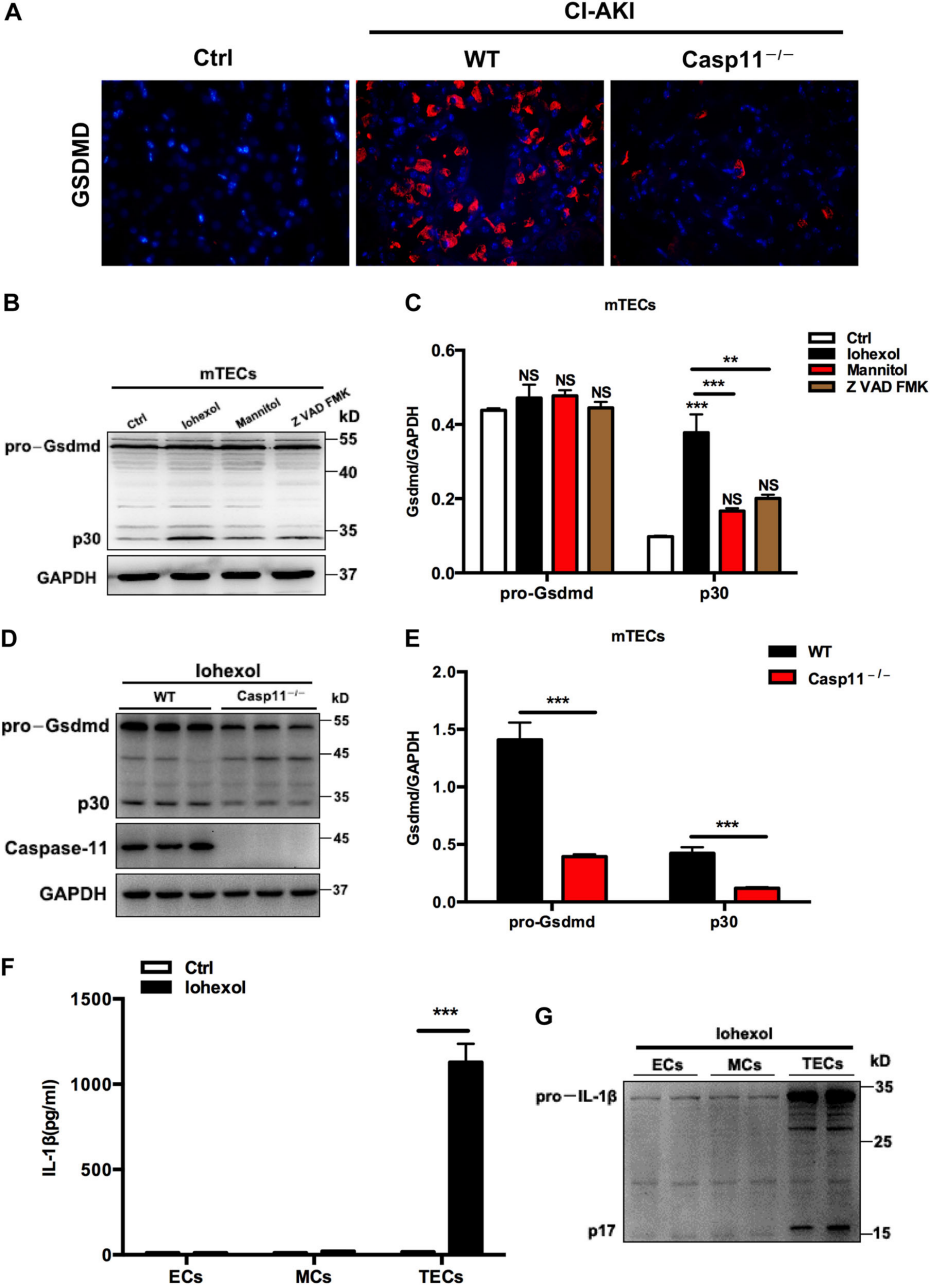
Renal tubular epithelial caspase-11 activation is required for generation of mature Gsdmd. **a** Representative images (three visual fields for each tissue analyzed) of immunolabelling for Gsdmd and the percentage of nuclear accumulation in WT and Casp11^–/–^ mice (*n* = 5). **b**, **c** TECs were isolated from kidneys and immunoblot analysis of the pore-forming mediator of pyroptosis. Gsdmd showed that iohexol (20 mg/ml) or isosmotic mannitol markedly increased the formation of the active, cleaved Gsdmd p30 protein in mouse TECs, which was blocked by the pan-caspase inhibitor Z-VAD-FMK. **d**, **e** Gsdmd cleavage was suppressed in TECs of Casp11^–/–^ mice (*n* = 6) following iohexol (20 mg/ml) or isosmotic mannitol challenge for 72 h. **f** Renal TECs, ECs, and MCs were exposed to iohexol (20 mg/ml) or isosmotic mannitol, and IL-1β secretion was measured. **g** Western blotting. Iohexol induced pro-IL-1β and mature IL-1β expression only in TECs. Ctrl control. **P < 0.01, ***P < 0.001 vs. control or as indicated. Data are shown as mean ± SEM. Statistics obtained from ANOVA

The pore-forming pyroptosis perforin Gsdmd is a critical target of caspase-11, which is cleaved into its active form by caspase-11 and forms pores promoting cell swelling and lytic cell death^13,14^. Immunofluorescence staining demonstrated specifically Gsdmd + TECs were significantly increased by iohexol treatment, but the response was abrogated in Casp11^–/–^ mice (Fig. 6a). To further address the role of caspase-11 in Gsdmd cleavage, we isolated TECs from Casp11^–/–^ and WT mice, and observed that iohexol markedly increased formation of the active, cleaved Gsdmd p30 protein in TECs from WT mice (Fig. 6b, c). Moreover, Gsdmd cleavage was inhibited in mouse TECs of Casp11^–/–^ mice following iohexol challenge (Fig. 6d, e). To determine whether iohexol have a similar potential to activate other renal parenchymal cells, we stimulated primary renal TECs, endothelial cells (ECs), and mesangial cells (MCs) with iohexol. Iohexol induced IL-1β secretion in renal TECs, whereas both ECs and MCs did not respond to the stimulation (Fig. 6f, g). Western blotting confirmed this finding and also showed that iohexol stimulation only induced pro-IL-1β and mature IL-1β expression in renal TECs (Fig. 6g). Hence, iohexol induced secretion of mature IL-1β only in renal TECs and not in other renal parenchymal cells (Fig. 6f).

## Discussion

Pyroptosis mediated by inflammatory caspases has an important role in innate immune defense against pathogen-associated molecular patterns and DAMPs^26,27^. It is characterized by lytic cell death triggered by activation of caspases 4/5 in human and caspase-11 in mice, which in turn leads to Gsdmd-induced pore formation and cleavage of inflammatory cytokine IL-1β, unlike the other forms of programmed cell death^4,7^. As unfettered pyroptosis can induce tissue injury, targeting caspase-mediated pyroptosis in specific cell types may be a useful strategy for limiting inflammatory tissue damage and organ failure without compromising host defense. The present study investigated the role of pyroptosis specifically in TECs and the potential of targeting epithelial pyroptosis in CI-AKI. TECs demonstrated lytic cell death, activation of Gsdmd, and release of the proinflammatory cytokine IL-1β, which are dependent on caspase-4/5 in human TECs and caspase-11 in mouse TECs. We demonstrated on the basis of genetic caspase-11 deletion studies in mice the requisite role of epithelial pyroptosis in the mechanism of CI-AKI. We showed that contrast media mediated pyroptosis via caspase-11 in TECs, which was required for the development of CI-AKI. Contrast media (iohexol) breached the epithelial plasma membrane and entered epithelial cytoplasm and then induced caspase-4/5/11 activation, which triggered pyroptosis via Gsdmd cleavage (Fig. 6). We demonstrated that widespread lysis of TECs resulted in severe disruption of tubules and the epithelial cells, release of proinflammatory cytokines, and increase of SCr and BUN levels, which are all hallmarks of AKI^1,3,5^.

Caspase-11-dependent pyroptosis was well described in macrophages and dendritic cells^13–15,26,28,29^; however, the critical role of this non-canonical cell death in TECs has not been established. Here we demonstrated pyroptosis is in caspase-4/5/11-dependent epithelial cell lysis. Distinctive features included rupture of the plasma membrane and release of lactate dehydrogenase (LDH), and maturation and release of IL-1β, as well as cleavage of Gsdmd, the pyroptosis executor^13,14,30^. We demonstrated that deletion of caspase-11 prevented contrast-induced damage in tubules and epithelial cells and markedly decreased the SCr and BUN levels. Thus, caspase-11 deficiency in mice abrogated CI-AKI, establishing the central role of caspase-11 expressed in TECs in the mechanism of CI-AKI.

Contrast-induced pyroptosis in TECs also highlights its role as an immune-regulating cell and regulator of innate immunity in AKI. TECs are the direct point of contact for various toxic drugs such as contrast media and thus DAMPs are released from intracellular compartments when the destructive stimulus occurs. It is now clear that the innate immune system can also respond to non-microbial stimuli, particularly to intracellular molecules that are released by necrotic cells^4,31–33^. However, DAMP signaling can also trigger inappropriate inflammation in sterile types of injury and thereby contribute to unnecessary organ damage and dysfunction. Hence, the TECs should be referred to not only as an “effector cell” regulating release of cytokines such as IL-18, tumor necrosis factor-α, and maintaining fluid balance, but also as a cell capable of sensing toxic stimulus and activating pyroptosis^34^. Even though our studies establish an essential role for epithelial caspase-11-mediated pyroptosis in CI-AKI, it remains unknown how the contrast media enters the cytoplasm or breaches the cell membrane and activates caspase-11 to trigger TECs lytic death.

Nowadays, the most common types of contrast agents used for intravascular injection are either iso-osmolar (approximately 290 mOsm/kg) (iodixanol) or low-osmolar (700 to 850 mOsm/kg) non-ionic monomers (iohexol)^35^. Osmolality is a major factor to be considered in clinical practice. In our study, we used the non-ionic monomer iohexol and isosmotic mannitol as an osmolality control to evaluate contrast media and osmolality itself in the epithelial pyroptosis. Our findings show that iohexol-induced pyroptosis in TECs was partially due to its osmolality, which also induced release of proinflammatory cytokine mature IL-1β, and cell lysis determined by LDH release. More importantly, we observed lower effect of osmolality itself in epithelial pyroptosis when compared with iohexol, suggesting that other contents of iohexol also contribute to the epithelial pyroptosis and need to be further studied. Our studies suggest that epithelial pyroptosis serve as a target for treatment of CI-AKI, but it also raise the intriguing question of whether contrast media themselves can initiate other signaling events that perpetuate or amplify epithelial cell injury and CI-AKI.

Through genetic screening, Gsdmd has been identified as a key mediator of pore formation of cells undergoing pyroptosis^13–15,17,30^. Gsdmd is a critical target of caspase-11, which cleaves it to generate N-terminal fragments (also known as p30), the “executioners” of pyroptosis^13,14,18^. Overexpression of cleaved Gsdmd alone induced pyroptosis independently of caspase-11 in vitro^14^. Furthermore, macrophages lacking functional Gsdmd were resistant to lytic death in response to intracellular lipopolysaccharide (LPS), whereas deletion of caspase-1 continued to exhibit Gsdmd cleavage^13,14^. However, the interrelationship of caspase-1 and caspase-11, and the mechanisms involved in epithelial pyroptosis and related CI-AKI remain unclear. Thus, studies are needed to clarify the role of caspase-1 or the overlapping roles of caspase-1 and caspase-11 in epithelial pyroptosis. Even though our studies establish an essential role for epithelial caspase-11 in mediating pyroptosis of the epithelium and CI-AKI, it remains unknown whether this process also activates the epithelial inflammasome in a non-canonical manner similar to that reported in phagocytic cells^13,14^.

In summary, we demonstrate that pyroptosis of TECs has a fundamental role in host-defense and immune surveillance functions of the toxic contrast media. Increased expression of caspase-4/5 in human and TECs is required for activation of epithelial pyroptosis; they thereby contribute to the development of CI-AKI. Thus, therapeutic strategies aimed at preventing epithelial pyroptosis may preserve tubular epithelial integrity and be beneficial in treating CI-AKI.

## Methods

### Materials

Human renal TECs and epithelial cell medium were obtained from ScienCell. Iohexol was obtained from GE Healthcare and Mannitol was obtained from Sigma. Mouse anti-rabbit IgG and Mouse anti-rat IgG antibodies were purchased from Cell Signaling Technology. We obtained antibodies targeting caspase-11 (Sigma, clone 17D9), caspase-4 (Abcam., ab40887), caspase-5 (Abcam, ab25898), IL-1β (R&D Systems, AF-401-NA), GAPDH (Cell Signaling Technology, #2118), Gsdmd (Novus Biologicals, NBP2-33422), and cytokeratin-18 (Abcam, ab668). Pan-caspase inhibitor Z-VAD-FMK was obtained from R&D Systems.

### Mice

Mice were housed under specific pathogen-free conditions at Slac Laboratory Animal Center in Shanghai and were randomly assigned to experimental animal groups. All animal experiments were conducted following the Animal Protocol Committee of Shanghai Jiao Tong University and approved by the Animal Care Committee at the Ren Ji Hospital, School of Medicine, Shanghai Jiao Tong University. Casp11^–/–^ mice were on a C57BL/6 background and obtained from the Jackson Laboratory. C57BL/6N mice acquired from Slac Laboratory Animal Center were used as WT controls. Male mice, 6–8 weeks old, were used for all experiments.

### CI-AKI in mice

Methods for CI-AKI model were previously described^20^. Briefly, male mice (aged 6–8 weeks) were anesthetized with isoflurane and mouse iohexol-induced AKI was created by ablating the right kidney and depriving the mouse of water for 24 h 3 weeks after the surgery, then followed by injection of furosemide (10 μl/g) via tail vein (model). Twenty minutes later, after furosemide injection, the mouse was administrated with iohexol (10 μl/g) or NS via tail vein. For immunohistochemistry and H&E staining, the mice were perfused with 4% paraformaldehyde (PFA) via the left ventricle and kidney paraffin-embedded at 6 mm thickness were used. For immunoblot analysis and real-time PCR analysis, the renal cortex and the outer strips of the medulla were snap-frozen in liquid nitrogen for total cellular protein isolation and RNA extraction.

### TEC culture

The primary mouse renal TECs were isolated as previously described^36^. In brief, 4–6 weeks old Casp11^–/–^ or WT mice were used for renal cell extraction. Decapsulated mouse kidneys were minced and passed through 80-mesh and 100-mesh sieves. The upper mixture was collected and digested with 1 mg/ml collagenase at 37 °C for 20 min. Terminating with fetal bovine serum and the digest mixture was centrifuged at 1000 × *g* for 5 min, washed, and plated into 6 cm cell culture dishes. The renal tubular cells were cultured under sterile conditions at 37 °C and 5% CO_2_ in conditioned medium in epithelial cell medium-animal (ScienCell) with 2% fetal bovine serum (ScienCell), 1% epithelial cell growth supplement-animal (ScienCell), and 1% penicillin/streptomycin solution (ScienCell). Medium was changed 48 h after initial plating. For the assessment of the purity of the mouse TECs population, the cells were grown in the 12-well plates to confluency, then fixed in ice-cold 4% PFA, stained for the markers of the TECs cytokeratin-18 and 4′,6-diamidino-2-phenylindole for nuclei and analyzed with fluorescence microscopy. Isolated mouse TECs were more than 90% pure. Confluent cells were treatment with iohexol as experiment design. Primary mouse renal ECs and MCs were isolated as previously described^37^.

Primary human renal TECs were purchased from ScienCell and cultured in epithelial cell medium supplemented with 2% fetal bovine serum (ScienCell), 1% epithelial cell growth supplement (ScienCell), and 1% penicillin/streptomycin solution (ScienCell). Adherent human TECs were then treated with iohexol and isosmotic mannitol in separate experiments. Experiments were performed in triplicate.

### Immunoblotting

For immunoblotting, cells were lysed with RIPA buffer. Supernatants were precipitated with 7.2% trichloroacetic acid plus 0.15% sodium cholate. Protein concentrations were determined using Pierce BCA Protein Assay Kit (Thermo Fisher). Proteins (20–50 μg) were boiled, separated on SDS-polyacrylamide gel electrophoresis, and transferred onto polyvinylidene difluoride membranes (Bio-Rad). Membranes were blocked with 5% bovine serum albumin in TBS with 0.1% Tween 20 (Sigma-Aldrich) for 1 h at room temperature and incubated overnight at 4 °C with primary antibodies. Densitometry analysis was performed using ImageJ software (U.S. National Institute of Health, Bethesda, MD) and the data were normalized against GAPDH.

### Histology and histopathology

Kidneys were fixed in 4% PFA and then embedded in 10% paraffin. Sections (5 μm thick) were cut for H&E staining, for further microscopic analysis. The tubular injury score was calculated according to the following grades: grade 0, normal; grade 1, <25%; grade 2, 25–49%; grade 3, 50–74%; grade 4, ≥75%.

### Immunohistochemistry

Paraffin-embedded sections (5 μm thick) were deparaffinized and rehydrated. Then antigen was retrieved at 98 °C for 10 min in 10 mM citrate buffer with pH 6 and washed with phosphate-buffered saline (PBS) for 15 min, and then the sections were treated with blocking buffer containing 5% bovine serum albumin for 30 min at room temperature before the overnight incubation with the primary antibodies. The following antibodies were used: anti-rat Caspase-11 (Sigma, clone 17D9, 1:400) and anti-mouse IL-1β (R&D Systems, AF-401-NA, 1:300). After washing with PBS for three times, the secondary antibody was added and counterstaining hematoxylin was performed, and DAB positivity was analyzed in six visual fields at ×200 magnification. The Caspase-11-positive area and IL-1β-positive area were measured using ImageJ (U.S. National Institute of Health).

### Quantitative real-time PCR

Gene expression for IL-1β, KIM-1, IL-18, and IL-6 was assessed by quantitative real-time PCR. Cellular or kidney RNA was extracted using RNAiso Plus kit (Takara, Dalian, China) according to the manufacturer’s instructions. One microgram of total RNA was reverse-transcribed to cDNA using PrimeScript RT Master Mix kit (Takara). Real-time PCR reactions were performed in triplicate using SYBR Premix Ex Taq II (Tli RNaseH Plus, Takara) and analyzed with a Light Cycler 480 (Roche). All primers were purchased from Takara. Relative changes in mRNA were calculated using the ΔΔCt method^38^ and standardized to housekeeping gene GAPDH.

Mouse genes and primers:

*Il1β*, forward, 5′-TCCAGGATGAGGACATGAGCAC-3′,

reverse, 5′-GAACGTCACACACCAGCAGGTTA-3′;

*Kim1*, forward, 5′-CCTTGTGAGCACCGTGGCTA-3′,

reverse, 5′-TGTTGTCTTCAGCTCGGGAATG −3′;

*Il18*, forward, 5′-ACCTCCAGCATCAGGACAAAG-3′,

reverse, 5′-TGTACAGTGAAGTCGGCCAAAG-3′;

*Il6*, forward, 5′-ACTTCCATCCAGTTGCCTTCTT-3′,

reverse, 5′-GCACAACTCTTTTCTCATTTCCAC-3′.

Human genes and primers:

*IL1β*, forward, 5′-CCAGGGACAGGATATGGAGCA-3′,

reverse, 5′-TTCAACACGCAGGACAGGTACAG-3′.

### Immunofluorescence

Deparaffinized and rehydrated paraffin-embedded sections (5 μm thick) were prepared for antigen retrieval at 98 °C for 10 min in 10 mM citrate buffer with pH 6. Then the sections were washed with PBS for 15 min and treated with blocking buffer containing 5% horse serum in PBS for 30 min at room temperature before the overnight incubation with the primary antibodies. Secondary Cy3-labeled antibodies were incubated with kidney sections for 1 h at room temperature. Negative controls for immunofluorescence staining were conducted using 5% horse serum instead of primary antibody. Images were analyzed by computerized digital image analysis (AnalySIS, Soft Imaging System).

### ELISA and LDH assay

Human TECs were cultured overnight in 96-well plates before treated with iohexol or isosmotic mannitol for 16 h. ELISA assays (enzyme-linked immunosorbent assays) were used to measure IL-1β (R&D Systems) in culture supernatants. A CytoTox 96 Non-Radioactive Cytotoxicity Assay (Promega) measured cell death according to the manufacturer’s instructions.

### Caspase-4/5 knockdown

Human TECs were transfected with 30 pM siRNA according to the manufacturer’s instructions (Life Technology).

### Statistics

Data were analyzed by two-tailed unpaired Student’s *t*-test for comparisons of two groups or two-way analysis of variance of the repeated experiments followed by the Tukey’s post hoc pairwise multiple comparisons when appropriate with Prism 6 (GraphPad). *P* < 0.05 was considered significant. For all bar graphs, the mean ± SEM is plotted. All in vitro experiments were repeated at least three times unless otherwise indicated.
